# Association between the severity of periodontitis, COVID-19, C-reactive protein and interleukin-6 levels in hospitalized patients: a case‒control study

**DOI:** 10.1186/s12903-023-03270-x

**Published:** 2023-08-11

**Authors:** Janet Moradi Haghgoo, Parviz Torkzaban, Maryam Farhadian, Sayed Ali Moosavi Sedeh

**Affiliations:** 1 Department of Periodontics, School of Dentistry, Hamadan University of Medical Sciences, Hamadan, Iran; 2 Department of Biostatistics, School of Public Health and Research Center for Health Sciences, Hamadan University of Medical Sciences, Hamadan, Iran

**Keywords:** Periodontitis, COVID-19, C-Reactive Protein, CRP, Interleukin-6, IL-6, Severity

## Abstract

**Background:**

The COVID-19 pandemic is perhaps one of the most important events of the 21^st^ century. Periodontitis is one of the most prevalent diseases of the oral cavity. Due to possible pathways of interaction between these two diseases, we investigated their association.

**Methods:**

The study population consisted of hospitalized patients with established COVID-19 diagnoses. Patients with mild to moderate COVID-19 were considered controls, while cases had severe to critical COVID-19. Periodontal examination and serum and saliva sampling were performed for each patient. Relevant medical data were extracted from patients’ hospital files.

**Results:**

Of the enrolled patients, 122 were included in the statistical analyses. The severity of periodontitis was directly and significantly correlated with the severity of COVID-19 (*P* < 0.001). Patients with generalized stage III or IV periodontitis displayed an adjusted odds ratio of 4.24 for severe to critical COVID-19. Salivary and serum interleukin-6 levels were significantly associated with COVID-19 severity (*P* values: 0.002 and 0.004, respectively). Hospitalization length was significantly associated with the severity of periodontitis (*P* = 0.004). Clinical attachment level and gingival index were associated with increased odds for adverse events (*P* values: 0.004 and 0.035, respectively), while number of remaining teeth was associated with decreased odds for adverse events (*P* = 0.023).

**Conclusions:**

This study showed that the severity of periodontitis is associated with the severity of COVID-19. This association might manifest as increased odds of adverse events. COVID-19 severity was associated with higher levels of salivary and serum interleukin-6 levels.

## Background

Some members of the Coronaviridae family of viruses naturally transfer between humans and cause mild upper respiratory tract infections with manifestations similar to the common cold. Conversely, some members of this family reside within the animal kingdom and cause more severe manifestations when they infect humans [1, 2]. COVID-19, caused by SARS-CoV-2, is suspected to be such a case [3]. Initially taking the world by storm and being declared a pandemic [4], COVID-19 has caused over 768 million confirmed cases and over 6.9 million deaths [5].

The cell entry receptors for this virus are angiotensin converting enzyme 2 (ACE2) and CD147 [6, 7]. Immune-system disturbances caused by the virus [8] pave the way for the disease to become severe/critical in 19% of cases [9]. These disturbances include viroporin-mediated cytokine release [10], antibody-dependent enhancement [11], and hyperinflammation leading to cytokine storm [8, 12]. Comorbidities, especially those with a chronic inflammatory nature (such as diabetes), further complicate the disease course and may significantly increase the odds for more severe forms of COVID-19 [13]. Among the comorbidities that may affect COVID-19 severity is periodontitis.

Periodontitis is the 6^th^ most prevalent human disease [14], and approximately 50% of the world’s population suffers from various degrees of periodontitis [15]. Simply put, periodontitis is a chronic, uncontrolled inflammation in response to the dysbiotic microbiome and biofilm in the gingival sulcus [16]. It is characterized by the continuous loss of supporting structures of the tooth, eventually causing tooth loss [17].

The periodontium contains the receptors and proteases required for SARS-CoV-2 entry [18], and SARS-CoV-2 has also been detected in gingival sulcus fluid [19]. Additionally, it has been documented that periodontitis increases systemic inflammation markers, such as interleukin-6 (IL-6) and C-reactive protein (CRP) [20]. Furthermore, it has been established that periodontitis has significant effects on systemic health; for example, diabetic patients will experience worse glycemic control with untreated periodontitis, and proper management of periodontitis helps with glycemic control in diabetic patients [21].

Both periodontitis and COVID-19 increase systemic inflammation levels and associated markers, such as IL-6 and CRP [20, 22–27]. In COVID-19 patients, IL-6 and CRP levels are also disease severity indicators and predict patient survival [25, 26]. Considering the possible direct interaction of COVID-19 with the periodontium, their interactions via inflammatory signaling molecules, and their effects on comorbidities and systemic health, it only seemed logical that the association between these two diseases should be investigated. Therefore, in this study, we evaluated the association between the severity of periodontitis, COVID-19, IL-6, and CRP. To objectively assess COVID-19 severity and be able to evaluate more severe forms, we decided to conduct this study in a controlled hospital setting on hospitalized patients.

## Methods

### Study design

Hospitalized patients with a confirmed COVID-19 diagnosis who matched the inclusion criteria (explained in the next sections) were enrolled in the study. During the hospitalization of each patient, a detailed periodontal examination was done (discussed in the “ Periodontal Examination” section), and a sample was taken from their serum and saliva (discussed in the “Serum and saliva sampling” section). After the end of the hospitalization, medical data were collected, and the COVID-19 severity assessment was done. Patients who matched the criteria for mild to moderate COVID-19 were considered controls, while patients who matched the criteria for severe to critical COVID-19 were considered the case group. Patients were then retrospectively investigated for the evaluation of the hypotheses.

The distribution of periodontal disease severity and CRP and IL-6 levels among the case and control groups was retrospectively evaluated to investigate any possible association between COVID-19 and periodontitis (i.e., the primary exposure variable was periodontitis severity and the primary outcome variable was COVID-19 severity). The primary objective was to evaluate the association between periodontitis severity, COVID-19 severity, and levels of IL-6 and CRP. Secondary objectives included evaluating the associations between background characteristics and disease severity, between periodontitis severity and hospitalization length, and between periodontal parameters and adverse events. Analyses of hematological tests, such as complete blood count, electrolytes, coagulation, and other tests, are performed in the second part of this study [28]. This study follows the STROBE guidelines for reporting its results.

### Setting

This part of the study shares its setting with the other part [28]. The details of the study setting were the following: Enrollment and examinations were done at a hospital with dedicated COVID-19 ward and intensive care unit (ICU). Patient enrollment was done by a single researcher at each hospital visit, which were scheduled randomly. At each hospital visit, all patient files in the COVID-19 ward were screened by this researcher, and patients who had an established COVID-19 diagnosis and matched the inclusion criteria were approached for written informed consent and subsequently enrolled in the study. Patients were assured that the data associated with them would be anonymized and could not be used to identify them, they would remain anonymous, and their COVID-19 treatment course would not be impacted by study participation. An extensive periodontal examination, in addition to saliva and serum sampling, was done for each enrolled patient, and they were followed for changes in their COVID-19 severity during their hospitalization.

### Participants

Patient enrollment was done from December 2021 to October 2022. The study population consisted of hospitalized patients with an established definitive COVID-19 diagnosis (positive SARS-CoV-2 PCR test and clinical COVID-19 symptoms). This part of the study shares its inclusion and exclusion criteria with the other part of the study:

- **Inclusion criteria** [28]: patients over the age of 18 years; no history of any genetic syndrome causing periodontitis as its manifestation (e.g., Papillon-Lefèvre syndrome, Leukocyte Adhesion Deficiency, Chédiak-Higashi syndrome,…); no uncontrolled metabolic disease; no active neoplasm; no history of chemotherapy within the last 3 months; no history of radiotherapy within the last 6 months; no immunosuppressive therapy; not pregnant; having either a healthy periodontium or mild localized gingivitis or generalized periodontitis.
- **Exclusion criteria** [28]: metabolic disease proven to be uncontrolled (determined by attending physician’s notes); periodontal examination not clinically possible; necrotizing periodontitis; fewer than 10 teeth remaining; superinfection with other infectious agents; diagnosis of a new neoplasm or relapse of an old neoplasm; history of intravenous drug abuse; history of comprehensive periodontal treatment within the last 6 months; and withdrawal from participation.

### Periodontal examination

A single periodontist was responsible for all periodontal examinations. Periodontal examination was performed using a mirror and a standard periodontal probe with William’s markings (Hu-Friedy, Chicago, Illinois, US). Three periodontal indices were measured: the clinical attachment level (CAL), the modified sulcus bleeding index (MSBI), and the gingival index (GI). The number of remaining teeth (excluding third molars) was also recorded.

To calculate the CAL, the distance from the base of the periodontal pocket to the cementoenamel junction (CEJ) was measured using a light (0.25 N) probing force. The criteria originally proposed by Löe & Silness [29] were used to calculate the GI: 0 = normal gingiva without inflammation; 1 = mild inflammation, slight color changes, and slight edema; 2 = moderate inflammation, redness, edema, and bleeding on probing; 3 = severe inflammation, marked erythema, ulceration, and spontaneous bleeding. The MSBI was used as a measure to quantify the degree of bleeding on probing and was calculated using the criteria proposed by Mombelli et al. [30]: 0 = no bleeding; 1 = isolated points of bleeding; 2 = a confluent line of bleeding on the gingival margin; and 3 = heavy or profuse bleeding. These indices were measured around every tooth (excluding third molars) at six sites (mesiobuccal, mid-buccal, distobuccal, distolingual, mid-lingual, and mesiolingual). The highest measurement for each index in each sextant was recorded.

#### Serum and saliva sampling

Serum sample collection was performed via routine antecubital venipuncture performed by a trained registered nurse. Collected blood was transferred to a serum separator tube (Separmed® test tube, F.L. Medical s.r.l. unipersonale, Torreglia, Italy) and centrifuged at 2000 g for 10 min. Separated serum samples were transferred to plain polyethylene test tubes and stored in a -70 °C refrigerator until analysis.

Saliva samples were collected using chilled (4 °C) plain polyethylene test tubes (Plain 10 ml Test Tubes, Ayset Tıbbi Ürünler San. A. S., Adana, Turkey). Between 9 and 11 a.m., when at least 2 h had passed since the last meal, nonstimulated whole saliva was collected from each patient using simple expectoration. Collected saliva samples were centrifuged at 1100 g for 5 min. An aliquot was separated and transferred to a -70 °C refrigerator until analysis.

To measure CRP and IL-6 levels, saliva and serum samples were thawed and analyzed using an automatic analyzer (IMMULITE® 2000, Siemens Healthineers, Erlangen, Germany; RRID:SCR_020514).

#### Medical data collection

Relevant sections in each patient’s hospital files were saved in image formats upon their discharge, in addition to their charts, lab results, and electronic records. After the conclusion of the sampling and the discharge of the last enrolled patient, all enrolled patients were followed for their discharge status, overall outcomes, missing data, and any further changes using the hospital’s information system.

To distinguish the condition of the patient in a short time frame before being discharged from the hospital from the entire hospitalization period, the last 3 records (L3A) on patients’ files/charts were used for suitable parameters, in addition to the mean, maximum, and minimum. As explained in the other part of the study [28], the following data were collected/calculated: age, sex, hospitalization length (start and discharge dates), intensive care, body temperature (last 3 records’ average, maximum), respiratory rate (last 3 records’ average, maximum), worst respiratory status, the maximum, mean, minimum, L3A, and baseline oxygen saturation with passive or assisted oxygenation, blood pressure (diastolic and systolic L3A), lung involvement in CT scan (appearance and extent), significant medical history and comorbidities, smoking status and history, organ involvement, medications before COVID-19 hospitalization, and major treatment medications during hospitalization.

#### Definitions and groups

The definitions and grouping were similar to those in the other part of the study, which are discussed in detail below [28]:

A periodontitis case was defined as the presence of either two nonadjacent labiolingual sites with a CAL of 3 mm or more accompanied by pocket formation, or two nonadjacent interproximal sites with detectable attachment loss [31]. The stage of periodontitis was determined based on the framework of the new classification of periodontal diseases [32]. Periodontal disease severity was divided into three categories [28]:

- Healthy Periodontium (HP): healthy periodontium, reduced but healthy periodontium, localized mild gingivitis
- Mild to moderate periodontitis (MP): generalized stage I or II periodontitis
- Severe periodontitis (SP): generalized stage III or IV periodontitis

To investigate the more extensive forms of periodontal disease and avoid including cases of incidental attachment loss, we included patients who had CAL in two sextants or more, and had signs of inflammation such as bleeding on probing and a gingival index higher than zero. Patients with CAL confined to a single sextant (localized cases) were not included in the study. The prominent factor for determining the periodontitis stage was the site with the greatest periodontal destruction, as described by Tonetti et al. [32], while the condition of other sites, in addition to other factors such as the number of remaining teeth, were also contributing factors.

COVID-19 associated organ involvement was defined as below [28]:

- Liver: Aspartate transaminase or alanine transaminase levels higher than 3 times their normal upper limit (NUL); bilirubin levels or phosphatase alkaline levels higher than 2 times their NUL [33].
- Kidney: ≥ 50% increase in creatinine levels compared to baseline in a 7-day period or ≥ 0.3 mg/dL increase in creatinine levels in a 48-h period [34].
- Hemato/Vascular: Deep Vein Thrombosis, Pulmonary Thromboembolism, Disseminated Intravascular Coagulation [35, 36]

COVID-19 severity grouping was done retrospectively using the following criteria based on a combination of WHO Guidelines [37] and the NHC of China’s classification [25]:

- Mild to moderate COVID-19 (MC): Flu-like Manifestations, Fever, Respiratory Symptoms
- Severe to critical COVID-19 (SC): Respiratory Distress, Respiratory Rate > 30, Passive Oxygenation 90% or below, Assisted Oxygenation 93% or below, ICU Transfer, Invasive Ventilation, 50% or more Radiographic Involvement, Shock, Multiorgan Failure, Sepsis

An adverse event was defined as the occurrence of any of the following: respiratory failure with subsequent need for invasive ventilation, ICU transfer, shock, multiorgan failure, sepsis, coma, or death.

#### Confounders and covariates

As in the other part of the study, all available data regarding confounders was collected whenever possible. The details are discussed below [28]: Data for age, sex, smoking, comorbidities, medications, body mass index (BMI), and socioeconomic status were collected. Smoking status was divided into three categories: current smokers, nonsmokers, and former smokers. We were not able to quantify smoking status in the form of pack-years or cigarettes per day since not enough standardized data were available. Enough data didn’t exist to reliably determine socioeconomic status or calculate BMI.

Based on all medical histories, comorbidities were divided into nine major categories [28]: diabetes mellitus, coronary artery disease (including angina, myocardial infarction, etc.), congestive heart failure, renal disease (including chronic kidney disease, end-stage renal disease, etc.), chronic liver disease (including non-alcoholic fatty liver disease, cirrhosis, etc.), respiratory disorders (including asthma, chronic obstructive pulmonary disease, bronchiectasis, etc.), autoimmune disorders (including arthritis rheumatoid, systemic lupus erythematous, multiple sclerosis, etc.), hypertension, and cancer in remission.

Medications were divided into three major groups: baseline-condition medications (such as medications for diabetes and hypertension), principal COVID-19 medications, and other medications (such as antiacids, analgesics, and laxatives). Principal COVID-19 medications included corticosteroids, anticoagulants and antiplatelets, antivirals (e.g., remdesivir), antibiotics, bronchodilators, and cytokine inhibitors (e.g., tocilizumab).

#### Bias reduction, sample size calculation, data analysis

This part of the study shares its design and sample pool with the other part of the study, and thus the details for bias reduction, sample size calculation, and confounder adjustment are similar to those in that part, which are listed below [28]:

Examination and data collection biases were reduced by blinding the periodontal examiner to the medical information of the patient and the medical file screener to the oral status of the patient. In other words, the periodontal examiner was not aware of the patient’s COVID-19 severity or the specific medical status, and the medical file screener was not aware of the patient’s periodontal status. At the conclusion of the study, all enrolled patients’ files were rechecked using the hospital’s information system in a single session to gather any new data and possibly acquire any missing information. All cases and controls were included in the analyses and no matching was performed.

As there was no similar published study at the time of the conception of this study, a previous study on the association between periodontitis and IL-6 levels [22] was used to calculate the sample size, since the association between periodontitis severity and IL-6 levels is being investigated here as well. Considering an alpha value of 5%, in order to reach a power of 80%, while assuming an expected mean difference of 0.7 pg/dL in the levels of IL-6 between different periodontitis severity groups and a variance of 0.8 pg/dL, a minimum sample size of 120 in total (average of 20 patients in each of the 6 subgroups) was required. Data analysis was performed using IBM SPSS Statistics v26 (IBM Corp., New York, USA; RRID: SCR_019096).

Regarding smoking status, since there was missing data and no statistically significant difference across COVID-19 or periodontal severity groups, it was excluded from the confounders adjusted for in the analyses. The same applied to the treatment medications, because no statistically significant difference was observed across COVID-19 or periodontal severity groups. There was not enough available data to reliably determine socioeconomic status or calculate body mass index for all patients. Therefore, age, sex, and comorbidities were determined to be the main confounders.

Sex and comorbidities were used as binary variables (female/male and no/yes, respectively) in the analyses, while age was used as a continuous variable. The severity assessments of COVID-19 and periodontitis were considered critical parameters and were available for all patients. The acceptable missing value threshold for analyses of noncritical parameters was set to a maximum of 20%.

The results for categorical variables were reported as frequency and percentage, and those for continuous variables were reported as the mean ± standard deviation. The Kolmogorov–Smirnov test was used to assess the normality of the distributions in the data. The chi-square test was used for the association analyses of categorical variables across severity groups. The independent sample t-test and one-way ANOVA were used for the analysis of continuous variables with normal distributions and comparisons across severity groups. To compare continuous variables with roughly normal distributions across severity groups while accounting for confounders and covariates and to compare the possible interaction between the two diseases, ANCOVA was used. Furthermore, to evaluate the association between COVID-19, periodontitis, IL-6, and CRP, and between adverse events and periodontal parameters, logistic regression analysis was used to calculate odds ratios with 95% confidence intervals while accounting for confounders and covariates. The Kruskal–Wallis test was used to compare hospitalization length between periodontitis severity groups since hospitalization length did not have a normal distribution.

## Results

### Participants

**Fig. 1:**
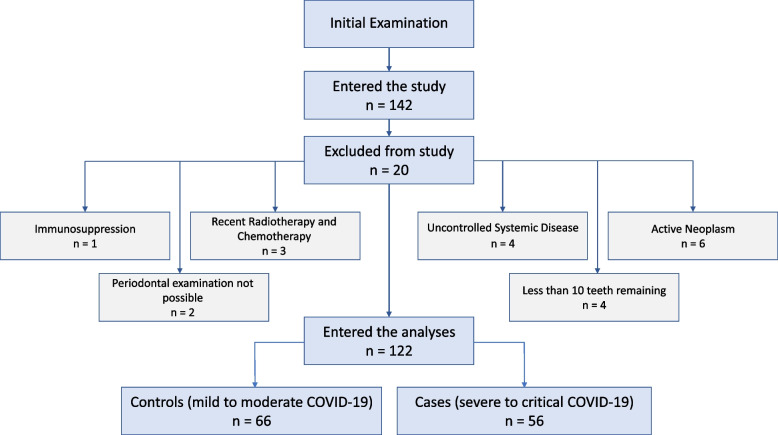
Enrollment flowchart

Initially, 142 patients were included in the study. Twenty patients were excluded from the study due to various reasons, upon the conclusion of follow-ups and a thorough examination of medical records. The diagram for study enrollment and the reasons for exclusion are presented in Fig. 1.

### General results

The mean, minimum, and maximum for age were 52.82 ± 13.34, 19, and 78 years, respectively, exhibiting a normal distribution. Of all patients, 48.4% (*n* = 59) were female. The minimum and maximum oxygen saturation with passive oxygenation for the entire hospitalization course were 86.16 ± 7.2% and 93.33 ± 3.71%, respectively. The most frequent appearance of lung involvement in CT scans was ground glass opacities, alone (*n* = 63, 52.9%) or combined with other appearances. Hypertension (33.6%, *n* = 41), diabetes mellitus (13.1%, *n* = 16), and chronic obstructive pulmonary disease (10.7%, *n* = 13) were the most common findings in medical histories. Of the patients with available smoking histories (*n* = 111), 70.3% (*n* = 78) had no smoking history. Nine (7.4%) patients had respiratory distress initially or during their hospitalization, and 27 (22.1%) of the patients required intensive care during their hospitalization. The liver was the most prevalent (13.9% of all patients, *n* = 17) organ with COVID-19 associated involvement. Adverse events occurred in 27.9% (*n* = 34) of patients, including three deaths. Length of stay (LOS) did not have a normal distribution, and the mean, median, and interquartile range for it were 10.24, 8, and 5 days, respectively. The general results can be found in Table 1.

**Table 1:** General medical data

**Variable**	**Subcategory**	**N**^**a**^	**Average ± SD**^**b**^	**Min**	**Max**	**Range**
**Temperature** (Celsius)	*Max*	110	37.23 ± 0.47	36.5	40	3.5
	*Min*	106	36.27 ± 0.46	34.2	37.4	3.2
	*L3A*^c^	106	36.69 ± 0.26	36.2	37.4	1.2
**Blood Pressure** (mmHg)	*Systolic L3A*	113	114.88 ± 12.35	93.33	150	56.7
	*Diastolic L3A*	113	71.86 ± 9.11	53.33	110	56.7
**Respiratory Rate** (per minute)	*Max*	107	21.07 ± 4.76	15	46	31
	*L3A*	105	18.01 ± 1.09	15	20.5	5.5
**Oxygen Saturation** (%)	*Assisted Oxygenation L3A*	81	95.28 ± 2.85	81	99.7	18.7
	*Passive Oxygenation Max*	101	93.33 ± 3.71	82	100	18
	*Passive Oxygenation Min*	106	86.18 ± 7.2	52	98	46
	*Passive Oxygenation L3A*	99	90.82 ± 4.83	73	98	25
	*Baseline*	111	85.44 ± 10.85	42	98	56
**CT Scan Lung Involvement**	*Extent (%)*	119	35.97 ± 16.83	5	75	70
**Variable**	**Subcategory**	**n**	**Count**	**Ratio (%)**^d^		
**CT Scan Lung Involvement Appearance**	*Ground Glass Opacities (GGO)*	119	63	52.9		
	*Consolidations*		7	5.9		
	*Atelectasis*		5	4.2		
	*Nodular*		4	3.4		
	*Embolic*		1	0.8		
	*GGO* + *Consolidations*		18	15.1		
	*GGO* + *Atelectasis*		9	7.6		
	*GGO* + *Embolic*		8	6.7		
	*GGO* + *Nodular*		4	3.4		
**Significant Medical History Items**	*Hypertension*	122	41	33.6		
	*Diabetes Mellitus*		16	13.1		
	*Neoplasm (in remission)*		14	11.5		
	*Chronic Obstructive Pulmonary Disease*		13	10.7		
	*End-Stage Renal disease*		10	8.2		
	*Coronary Artery Disease*		10	8.2		
	*Autoimmune Disorder*		10	8.2		
	*Hypothyroidism*		7	5.7		
	*Asthma*		6	4.9		
	*Cerebrovascular Accident*		5	4.1		
	*Chronic Kidney Disease*		4	3.3		
**Smoking**	*Yes*	111	30	27		
	*Former*		3	2.7		
	*No*		78	70.3		
**COVID-19 Associated Organ Involvement**	*Liver*	122	17	13.9		
	*Hemato/Vascular*		10	8.2		
	*Kidney*		7	5.7		
	*Multiple Organs*		8	6.5		
**ICU Admission**	*Yes*	122	27	22.1		
	*No*		95	77.9		
**Adverse Event**	*Yes*	122	34	27.9		
	*No*		88	72.1		
**COVID-19 Severity**	*Mild to Moderate*	122	66	54.1		
	*Severe to Critical*		56	45.9		
**Periodontitis Severity**	*Healthy / Localized Mild Gingivitis*	122	37	30.3		
	*Generalized Stage I or II*		42	34.4		
	*Generalized Stage III or IV*		43	35.2		

^a^Number of available data

^b^Standard Deviation

^c^Last three records’ average

^d^Percentage from available data(n)

### Association between COVID-19, periodontitis, age, and sex

There was a significant age difference between the COVID-19 and periodontitis groups. Independent t-test revealed that the SC group had a significantly higher average age than the MC group (*P* = 0.004). One-way ANOVA revealed a significant age difference between the three periodontitis groups (*P *< 0.001). Chi-square tests indicated no significant difference regarding sex between COVID-19 groups (*P* = 0.69), while there was a significant difference regarding sex between periodontitis groups (*P* = 0.01, Chi2 = 9.17). The results for these tests can be found in Table 2.

**Table 2:** Analyses of the association between COVID-19, periodontitis, sex, and age

**Disease**	**Group**	**Sex** **Frequency (%)**		**Chi2 Test**
		***Male***	***Female***	
**COVID-19**	*MC*	33 (52.4%)	33 (55.9%)	Chi2 = 0.155 *P* = 0.69
	*SC*	30 (47.6%)	26 (44.1%)	
**Periodontitis**	*HP*	14 (22.2%)	23 (39%)	Chi2 = 9.17 *P* = 0.01^*^
	*MP*	19 (30.2%)	23 (39%)	
	*SP*	30 (47.6%)	13 (22%)	
**Disease**	**Group**	**Age** **Mean + SD**^**a**^		**Test**
**COVID-19**	*MC*	49.71 ± 13.89		Independent t-test *P* = 0.004^*^
	*SC*	56.48 ± 11.76		
**Periodontitis**	*HP*	43.16 ± 12.2		ANOVA test *P* < 0.001^*^
	*MP*	54.98 ± 11.95		
	*SP*	59.02 ± 10.94		

^*^Statistically significant

^a^Standard deviation

### Association between COVID-19 and periodontitis

To test for a possible association between COVID-19 and periodontitis, the chi-square test was used. The results indicated that the severity of periodontitis and COVID-19 were significantly associated (*P* < 0.001). In fact, 74.4% (*n* = 32) of the SP group had severe to critical COVID-19. The results can be found in Table 3.

**Table 3:** Analysis of the association between COVID-19 and periodontitis severity

**Variable**		**Frequency (%)**				**Chi2 Test**
		**Periodontitis Severity**				
**COVID-19 Severity**	**Group**	*HP*	*MP*	*SP*	*Total*	Chi2 = 22.062 *P* < 0.001^*^
	*MC*	27 (73%)	28 (66.7%)	11 (25.6%)	66	
	*SC*	10 (27%)	14 (33.3%)	32 (74.4%)	56	
	*Total*	37	42	43	122	

^*^Statistically significant

To obtain odds ratios for periodontitis and COVID-19, while accounting for IL-6 and CRP levels and controlling for confounders, logistic regression was used. Compared to the HP group, the SP group had an adjusted odds ratio (AOR) of 4.24 for severe to critical COVID-19 (*P* = 0.047, 95% confidence interval: 1.020 – 17.683). IL-6 had significant association with severe COVID-19. Each unit increase in serum IL-6 levels would increase the odds of severe COVID-19 by 1.075 (*P* = 0.033, 95% confidence interval: 1.006 – 1.148). The results for crude odds ratios and other details can be found in Table 4.

**Table 4:** Logistic regression analysis for severe COVID-19

**Outcome**	**Parameter**	**Group**	**Crude Analysis**		**Adjusted Combined Analysis**^a^	
			***P* Value**	**Odds Ratio (95% CI**^b^**)**	***P* Value**	**Odds Ratio (95% CI)**
**Severe to Critical COVID-19**	Periodontitis Severity	*HP*^c^	-	1.0	-	1.0
		*MP*	0.544	1.350 (0.513 – 3.556)	0.528	1.551 (0.397 – 6.052)
		*SP*	< 0.001^*^	**7.855 (2.896 – 21.305)**	0.047^*^	**4.247 (1.020 – 17.683)**
	Serum IL-6	*-*	0.002^*^	**1.049 (1.017 – 1.082)**	0.033^*^	**1.075 (1.006 – 1.148)**
	Saliva IL-6	*-*	0.009^*^	**1.147 (1.035 – 1.272)**	-	-
	Serum CRP	*-*	0.097	1.014 (0.998 – 1.030)	0.495	0.991 (0.966 – 1.017)
	Saliva CRP	*-*	0.333	1.084 (0.920 – 1.277)	-	-
	Mean CAL	*-*	< 0.001^*^	**1.857 (1.424 – 2.423)**	-	-
	Mean GI	*-*	0.003^*^	**1.907 (1.242 – 2.926)**	-	-
	Mean MSBI	*-*	0.003^*^	**2.059 (1.277 – 3.322)**	**-**	**-**
	Remaining Teeth	*-*	< 0.001^*^	**0.807 (0.728 – 0.894)**	**-**	**-**
	Age	*-*	0.006^*^	**1.042 (1.012 – 1.073)**	0.109	1.042 (0.991 – 1.095)
	Sex	*Female*^c^	-	1.0	-	1.0
		*Male*	0.694	1.154 (0.565 – 2.354)	0.466	0.646 (0.2 – 2.088)
	Comorbidity	*No*^c^	-	1.0	-	1.0
		*Yes*	0.045^*^	**2.211 (1.016 – 4.809)**	0.370	1.732 (0.521 – 5.755)

*Statistically significant

^a^Variables in the combined model: COVID-19, Periodontitis, Serum IL-6, Serum CRP, Age, Sex, Comorbidity

^b^Confidence Interval

^c^Reference Group

### Association between COVID-19, periodontitis, and levels of CRP and IL-6

The ANCOVA adjusted for age, sex, and comorbidities revealed that none of the single nor combined models could account for changes seen in CRP levels across groups. On the other hand, the single models revealed that salivary and serum IL-6 levels had a significant association with COVID-19 (*P* values: < 0.001 and < 0.001, respectively) and periodontitis severity (*P* values: 0.002 and 0.006, respectively). The combined models revealed that while the interaction between periodontitis and COVID-19 did not have significant effect on IL-6 levels, the main effect of COVID-19 was significantly associated with serum and salivary IL-6 levels (*P* values: 0.002 and 0.004, respectively). These results can be found in Table 5.

**Table 5:** Analyses of the association between COVID-19, periodontitis, and inflammatory markers

**Marker**	**COVID-19 Severity**	**Mean ± Standard Deviation (number of available data)**				**ANCOVA**^**a**^	
		**Periodontitis Severity Group**				**Model**^**b**^	***P* Value**
		***HP***	***MP***	***SP***	***Total***		
**Serum CRP** (mg/dL)	*MC*	18.48 ± 23.52 (26)	27.77 ± 23.96 (22)	38.09 ± 24.11 (11)	25.60 ± 24.51 (59)	*COVID-19 Only*	0.063
						*Periodontitis Only*	0.098
	*SC*	26.43 ± 23.64 (7)	40.44 ± 25.06 (12)	32.75 ± 24.74 (24)	33.87 ± 24.53 (43)	Interaction Model:	
						*COVID-19*	0.152
	*Total*	20.17 ± 23.41 (33)	32.24 ± 24.75 (34)	34.43 ± 24.32 (35)	29.09 ± 24.74 (102)	*Periodontitis*	0.131
						*COVID-19* × *Perio*	0.225
**Saliva CRP** (mg/dL)	*MC*	0.83 ± 1.68 (26)	1.27 ± 2.69 (22)	2.45 ± 2.91 (11)	1.30 ± 2.38 (59)	*COVID-19 Only*	0.230
						*Periodontitis Only*	0.080
	*SC*	0.43 ± 1.13 (7)	2.33 ± 2.39 (12)	1.87 ± 2.66 (24)	1.77 ± 2.44 (43)	Interaction Model:	
						*COVID-19*	0.587
	*Total*	0.75 ± 1.58 (33)	1.65 ± 2.60 (34)	2.06 ± 2.71 (35)	1.50 ± 2.40 (102)	*Periodontitis*	0.063
						*COVID-19* × *Perio*	0.276
**Serum IL-6** (pg/dL)	*MC*	5.52 ± 8.71 (25)	9.82 ± 14.03 (25)	9.71 ± 8.81 (11)	8.04 ± 11.25 (61)	*COVID-19 Only*	< 0.001^*^
						*Periodontitis Only*	0.002^*^
	*SC*	9.53 ± 6.09 (7)	15.13 ± 14.28 (14)	23.52 ± 23.56 (25)	18.84 ± 19.74 (46)	Interaction Model:	
						*COVID-19*	0.002^*^
	*Total*	6.39 ± 8.29 (32)	11.73 ± 14.16 (39)	19.30 ± 21.08 (36)	12.68 ± 16.31 (107)	*Periodontitis*	0.101
						*COVID-19* × *Perio*	0.596
**Saliva IL-6** (pg/dL)	*MC*	1.37 ± 1.56 (25)	3.28 ± 5.39 (25)	3.11 ± 2.55 (11)	2.47 ± 3.81 (61)	*COVID-19 Only*	< 0.001^*^
						*Periodontitis Only*	0.006^*^
	*SC*	2.43 ± 1.52 (7)	5.35 ± 6.14 (14)	7.17 ± 8.22 (25)	5.90 ± 7.08 (46)	Interaction Model:	
						*COVID-19*	0.004^*^
	*Total*	1.60 ± 1.59 (32)	4.03 ± 5.68 (39)	5.93 ± 7.20 (36)	3.94 ± 5.69 (107)	*Periodontitis*	0.101
						*COVID-19* × *Perio*	0.744

^*^Statistically significant

^a^All analyses adjusted for age, sex, and comorbidities

^b^Three models were run: COVID-19 only, periodontitis only, and COVID-19 combined with periodontitis (the interaction model). In the interaction model, the main effects and the interaction are reported

### Association between adverse events and periodontal parameters

A significant association between CAL and adverse events (*P* = 0.004) and GI and adverse events (*P* = 0.035) was found using logistic regression. It was also revealed that the number of remaining teeth was inversely associated with adverse events (*P* = 0.023). No significant association was found regarding MSBI. The results are shown in Table 6.

**Table 6:** Logistic regression for periodontal parameters and adverse events

**Variable**	**Mean ± Standard Deviation**		**Logistic Regression**^**a**^	
	**Adverse Event**		***P* Value**	**Odds Ratio (95% Confidence Interval)**
	***No***	***Yes***		
**Mean Clinical Attachment Level**	1.61 ± 1.53	2.83 ± 1.53	0.004^*^	**1.649 (1.171 – 2.323)**
**Mean Gingival Index**	1.28 ± 0.88	1.83 ± 0.85	0.035^*^	**1.846 (1.045 – 3.261)**
**Mean Modified Sulcus Bleeding Index**	0.98 ± 0.77	1.44 ± 0.83	0.055	1.773 (0.988 – 3.181)
**Teeth Remaining**	24.15 ± 4.02	21.29 ± 4.22	0.023^*^	**0.875 (0.780 – 0.982)**

^*^Statistically significant

^a^All analyses adjusted for age, sex, and comorbidities

### Association between periodontitis and length of stay

To investigate the association between LOS and periodontitis, the Kruskal‒Wallis test was used. The results showed that periodontitis was significantly associated with LOS (*P* = 0.004). The results are shown in Table 7.

**Table 7:** Analysis of association between periodontitis and length of stay

**Periodontitis Group**	**Mean + Standard Deviation** (days)	**Median** (days)	**Interquartile Range** (days)	**Kruskal–Wallis test**
*HP*	8.0 ± 3.88	7.0	5.0	Kruskal–Wallis H = 11.245 *P* Value = 0.004^*^
*MP*	9.64 ± 7.95	7.5	5.0	
*SP*	12.74 ± 10.84	9.0	8.0	

^*^Statistically significant

## Discussion

### Key results and interpretation

In this study, we investigated the association between the severity of COVID-19 and the severity of periodontitis in hospitalized patients. We also investigated the possible links between these two diseases and other factors, such as age and sex. Age had a significant association with both COVID-19 and periodontitis severity. The SC group was approximately 7 years older than the MC group. This finding was similar to other studies [25, 26, 38, 39]. The explanation for this association is mainly a phenomenon called immunosenescence (i.e., immune system aging), which entails an increase in memory T cells and a decrease in naïve T cells. These cells also show less potential for proliferation but more potential for proinflammatory cytokine production [40]. More severe forms of periodontitis were also associated with increased age. The mean age of the HP group was 43.16 years, while that of the SP group was 59.02 years. The current consensus is that periodontitis is an age-associated disease, with an abundance of literature reporting similar findings [20, 41–44]. The reasons for this association are not yet entirely understood, but changes in innate and adaptive immunity have been at the forefront of possible causes [44].

Although there were more males in the SC group (53.6%, *n* = 30), the association between sex and COVID-19 severity did not reach statistical significance. Many studies have found that males are more predisposed to severe forms of COVID-19 [26, 45, 46]. Estrogen-mediated upregulation of ACE2 [47], which protects against the damaging effects of SARS-CoV-2-induced ACE2 downregulation, decreases the odds of females progressing toward more severe forms of COVID-19 [48]. Similarly, it has been documented that periodontitis is more prevalent in males [49], which is in line with our findings. A total of 47.6% (*n* = 30) of males were in the SP group, while only 22% (*n* = 13) of females were in the SP group. Reasons for this disparity are categorized into two major groups: biological differences between sexes (e.g., immune system, etc.), and social differences (e.g., socioeconomic factors, cultural attitudes, access to preventive and regular care, etc.) [50].

In this study, the association between the severity of periodontitis and COVID-19 was found to be statistically significant. This finding was similar to the study performed by Anand et al., which showed that severe forms of periodontitis had an odds ratio of 11.75 for COVID-19 infection [51]. Two other studies also found that severe forms of periodontitis were associated with higher odds of adverse events and complications and, hence, more severe forms of COVID-19 [52, 53]. We found that more severe forms of periodontitis had higher odds for severe COVID-19. The SP group had an adjusted odds ratio of 4.24 for severe COVID-19, compared to the HP group. Marouf et al. reported an AOR of 3.67 for adverse events, 3.54 for ICU admission, and 8.81 for death [52]. In agreement with the above findings, Gupta et al. reported an AOR of 7.45 for mechanical ventilation, 36.52 for hospital admission, and 14.58 for death in severe forms of periodontitis, compared to healthy patients [53]. Regarding periodontal parameters, we found significant associations between CAL and adverse events, and between GI and adverse events. Similar results were also found by Gupta et al. and Anand et al. [51, 53]; Gupta et al. found significant associations between bleeding on probing, mean probing depth and COVID-19 severity indicators such as hospitalization, need for assisted ventilation, and survival. Anand et al. found significant differences in pocket depth, CAL, and gingivitis between their case and control groups. It can be said that due to increases in systemic inflammation levels caused by severe forms of periodontitis, the COVID-19 course will be more likely to progress to a hyperinflammatory state and eventual cytokine storm. Subsequent to the cytokine storm, organ failure and medical complications [12] will necessitate intensive care and treatments in order to sustain the patient’s life.

Interleukin-6 is a major proinflammatory cytokine and one of the main signaling molecules responsible for the production of acute-phase proteins such as CRP [54]. It has been found that higher levels of this cytokine adversely affect the survival of COVID-19 patients [25, 26]. Compared to individuals with a healthy periodontium, patients with periodontitis show elevated levels of IL-6 [22, 24, 42]. In this study, we found a significant association between COVID-19 severity and IL-6 levels. C-reactive protein is a nonspecific acute-phase protein produced in the liver that is upregulated by inflammatory processes and may be an indicator of systemic inflammation and the severity of COVID-19 [55, 56]. In regards to periodontitis, it has been well documented that higher CRP levels are found in the saliva and serum of periodontitis patients compared to healthy individuals, and following treatment for periodontitis, CRP levels decrease [20, 22, 41]. The CRP increase in periodontitis is due to the fact that the chronic inflammation process of this disease increases systemic pro-inflammatory cytokines [20, 24], which would lead to the upregulation of inflammation markers such as CRP [57]. In our study, despite increased CRP levels in more severe forms of periodontitis and COVID-19, none of the adjusted statistical models could meaningfully account for the observed differences. In a review article, most of the reviewed studies reported increased CRP levels in severe forms of COVID-19 [56]. Higher levels of this biomarker are not only an indicator of the severity of COVID-19 but also an indicator of the prognosis of patients [26, 58].

IL-6 and CRP levels and their associations should be interpreted with caution. The most critical concern is that the timing of the serum and salivary sampling was not completely standardized. Even though all samples were collected between 9 and 11 a.m., one patient could be sampled on the 3rd hospitalization day, while another could be sampled on the 8th day. Coupled with the fact that the interval between the onset of symptoms and hospitalization was different between patients, this would mean different inflammatory timepoints in the disease course, resulting in different cytokine and marker profiles [59]. Another concern was the treatment regimens/medications for patients, especially immunomodulatory and immunosuppressive drugs. Although many patients received similar principal medications, and there was no significant difference regarding medications across groups, the dosages and response could have been different between patients, which may result in varying levels of host response, such as immune system activity. Furthermore, it has been established that there are genetic variations that may predispose individuals to higher risks for developing certain diseases or higher risks for progressing toward more severe conditions. In a recent meta-analysis by da Silva et al., two interleukins whose genetic variances could significantly increase the risk for chronic periodontitis were identified: IL-1α and IL-1β [60]. Specific genetic mutations of IL-1α were investigated in another review study and found to increase the severity of COVID-19 [61]. The IL-10 mutation rs1800872 has been found to increase the susceptibility of individuals to both periodontitis and COVID-19 [61–63]. These findings and future findings may further reveal the role of genetic variations in disease progression and may indicate that serum levels of interleukins alone may not be able to completely explain all findings.

The mean length of stay for all patients was 10.24 days. The mean LOS for the MC and SC groups were 7.06 and 13.98 days, respectively. The mean LOS for the HP, MP, and SP groups were 8, 9.64, and 12.74 days, respectively. The increase in LOS was significantly associated with the severity of periodontitis. There were no previous studies investigating the association between periodontitis and LOS in COVID-19 patients, but some studies have investigated the association between COVID-19 and LOS [39, 46, 64]. The main reason for this finding is postulated to be inadequate viral clearance, which at the time of seroconversion may lead to antibody-dependent enhancement and further worsening of the clinical course [8]. Periodontitis may be associated with hospitalization length due to the direct and indirect effects it has on COVID-19 severity.

There are many underlying pathways that link periodontitis with COVID-19, however, only a few have been mentioned thus far. While the genetic differences and cytokine connection have already been covered, other pathways also need to be taken into consideration. It has been demonstrated that SARS-CoV-2 entrance receptors, including ACE2 and CD147, are present in the oral cavity, particularly on epithelial cells [6, 7, 65], and therefore in the periodontium. It has also been shown that SARS-CoV-2 is present in the gingival sulcus [19] in detectable numbers high enough to be utilized as a reliable diagnostic test. Furthermore, there have been findings regarding the direct pathosis caused by SARS-CoV-2 in the periodontium, including direct fibroblast degeneration and senescence, hyperproliferation, and pathogenic fibrotic phenotypes [66]. Periodontitis can affect COVID-19 as well, with emerging evidence showing an association between the two diseases [51–53]. The elevation of systemic inflammation and the increase in cytokine levels in more severe forms of periodontitis [22, 24, 42, 67], many of which are directly associated with COVID-19 progression and adverse outcomes [26, 68, 69] is perhaps the most obvious way in which periodontitis can affect COVID-19. The second potential link might be between periodontitis and systemic comorbidities [70]. The effect of periodontitis on these comorbidities might indirectly influence COVID-19 infection due to the significant effects these comorbidities have on COVID-19 [71], many of which are already known [13, 46]. Finally, SARS-CoV-2 can independently replicate in the oral cavity and periodontium, establishing a reservoir in this region. The virus can then spread through the body via hematologic, digestive, or respiratory routes [71]. More information about the underlying pathways linking periodontitis and COVID-19 will become clear as more studies are conducted.

### Limitations and generalizability

A clinical diagnosis of periodontitis was made in this study, which is one of its strengths; nevertheless, one of its weaknesses was the lack of radiographic information to support the diagnosis. A hospital setting allowed us to study the more severe forms of COVID-19 in a more controlled environment. The retrospective assessment of COVID-19 severity might be prone to inaccuracies or inconsistencies in the documentation. We attempted to mitigate this issue by evaluating all available records during the entire hospitalization course, whether paper or electronic. As discussed previously, the sampling day was not standardized between patients, which might be a limitation for this study.

Studying hospitalized patients served as a double-edged sword. While it provided us with the means to study the more severe forms of COVID-19 and a more reliable clinical appraisal of the patient’s condition, it also resulted in a limitation on the generalizability of the findings of this study to the general COVID-19 population. The criteria for patient inclusion and exclusion allowed for better isolation of the interaction between periodontitis and COVID-19, but may also limit the generalization and extension of the results to the general population. Besides confounders such as age, sex, medications, smoking, and comorbidities, other confounders, such as BMI, socioeconomic status, and lifestyle, could not be accounted for, which may limit the validity of the results. More studies with possible standardizations on sampling and examination timings, with prospective longitudinal or controlled trial designs are required to further investigate the issues addressed in this article.

## Conclusions

The results of this study showed that there is an association between age and the severity of both COVID-19 and periodontitis. Sex was also associated with periodontitis severity. It was also shown that the severity of COVID-19 and periodontitis are associated, with higher periodontal parameters resulting in higher odds for adverse events. IL-6 levels were increased in severe forms of COVID-19. However, the levels of inflammatory markers should be interpreted with caution.

It is established that periodontitis affects systemic health and systemic inflammation levels, and this study portrays periodontitis as a possible factor associated with more severe forms of COVID-19. Based on this association, it may be plausible to integrate oral hygiene measures with or without professional debridement into the treatment plan for COVID-19 patients, which may result in reduced odds for COVID-19 progression and morbidities. Future research may reveal the exact underlying pathways that connect COVID-19 to periodontitis.

## Data Availability

The data that support the findings of this study are available from the corresponding author upon reasonable request. The data are not publicly available due to legal restrictions.

## Abbreviations

ACE2: Angiotensin Converting Enzyme 2
BOP: Bleeding on Probing
CAL: Clinical Attachment Level
CRP: C-Reactive Protein
GI: Gingival Index
HP: Healthy Periodontium Group
ICU: Intensive Care Unit
IL-6: Interleukin-6
LOS: Length of Stay
MC: Mild/Moderate COVID-19
MP: Mild/Moderate Periodontitis
MSBI: Modified Sulcus Bleeding Index
SC: Severe COVID-19
SP: Severe Periodontitis
